# Cytotoxic activity of the MK2 inhibitor CMPD1 in glioblastoma cells is independent of MK2

**DOI:** 10.1038/cddiscovery.2015.28

**Published:** 2015-09-07

**Authors:** FMS Gurgis, MC Åkerfeldt, B Heng, C Wong, S Adams, GJ Guillemin, TG Johns, M Chircop, L Munoz

**Affiliations:** 1 School of Medical Sciences, Department of Pharmacology, The University of Sydney, Sydney, NSW 2006, Australia; 2 Faculty of Medicine and Health Sciences, Macquarie University, NSW 2109, Australia; 3 Children’s Medical Research Institute, The University of Sydney, Sydney, NSW 2145, Australia; 4 MIMR-PHI Institute of Medical Research, 27–31 Wright Street, Clayton, VIC 3168, Australia; 5 Monash University, Wellington Road, Clayton, VIC 3800, Australia

## Abstract

MAPK-activated protein kinase 2 (MK2) is a checkpoint kinase involved in the DNA damage response. MK2 inhibition enhances the efficacy of chemotherapeutic agents; however, whether MK2 inhibition alone, without concurrent chemotherapy, would attenuate survival of cancer cells has not been investigated. CMPD1 is a widely used non-ATP competitive inhibitor that prevents MK2 phosphorylation. We employed CMPD1 together with MK2 knock-down and ATP-competitive MK2 inhibitor III (MK2i) in a panel of glioblastoma cells to assess whether MK2 inhibition could induce cancer cell death. While CMPD1 was effective at selective killing of cancer cells, MK2i and MK2 knock-down had no effect on viability of glioblastoma cells. CMPD1 treatment induced a significant G2/M arrest but MK2i-treated cells were only minimally arrested at G1 phase. Intriguingly, at doses that were cytotoxic to glioblastoma cells, CMPD1 did not inhibit phosphorylation of MK2 and of its downstream substrate Hsp27. These results suggest that CMPD1 exhibits cytotoxic activity independently of MK2 inhibition. Indeed, we identified tubulin as a primary target of the CMPD1 cytotoxic activity. This study demonstrates how functional and mechanistic studies with appropriate selection of test compounds, combining genetic knock-down and pharmacological inhibition, coordinating timing and dose levels enabled us to uncover the primary target of an MK2 inhibitor commonly used in the research community. Tubulin is emerging as one of the most common non-kinase targets for kinase inhibitors and we propose that potential tubulin-targeting activity should be assessed in preclinical pharmacology studies of all novel kinase inhibitors.

## Introduction

One hallmark of cancer cells is their ability to repair the DNA damage. In the event of DNA damage, the cell cycle is stalled at the G1/S, intra-S, and G2/M checkpoints. The cell-cycle arrests provide an opportunity for the cells to repair the DNA damage and survive. This mechanism also underlies the cancer resistance to DNA damaging chemotherapy.^1^ Checkpoint kinase 1/2 (Chk1/2) and Wee1 are examples of kinases regulating checkpoints in response to DNA damage. Numerous studies have demonstrated the therapeutic potential of inhibiting these kinases, resulting in sensitization to chemotherapeutic agents.^2–5^ Moreover, Chk1 and Wee1 inhibitors displayed single agent efficacy in cancer cells with specific defects in DNA repair or in cells that are dependent on a constitutive DNA damage response.^6–9^

p38 Mitogen-activated protein kinase (p38 MAPK) and its downstream substrate MAPK-activated protein kinase 2 (MK2) were identified as a third checkpoint pathway in addition to Chk1/2 and Wee1 signalling.^10–12^ In tumours lacking p53, inhibition of MK2 resulted in enhanced efficacy of chemotherapeutic agents.^13^ Mechanistic studies revealed that MK2 maintains G2/M checkpoint arrest until DNA damage is repaired through the post-transcriptional regulation of gene expression.^14^ In p53-proficient cancer cells, p38 MAPK–MK2 pathway has been implicated as a critical repressor of p53-driven apoptosis in response to doxorubicin and this is mediated by MK2-dependent phosphorylation of the apoptosis-antagonizing transcription factor.^15^ These studies highlight MK2 inhibition as a chemo-sensitizing strategy to treat both p53-deficient and p53-proficient cancers. However, whether MK2 inhibition alone, without concurrent chemotherapy, would reduce tumour cell proliferation has not been investigated.

p38 MAPK regulates activity of more than 60 substrates^16^ and its inhibition is therefore accompanied with unwanted side effects. MK2, being a downstream substrate with fewer signalling pathways, represents a potentially better therapeutic target. However, inhibiting MK2 with ATP-competitive inhibitors is challenging because of the high affinity of MK2 towards ATP.^17^ MK2 inhibitors, even if highly potent in biochemical *in vitro* assays, are weakly active in cells and *in vivo* due to the high competition with ATP. On the other hand, non-ATP competitive inhibitors offer the advantage of avoiding ATP competition and are currently under development. CMPD1 was developed as non-ATP-competitive inhibitor of p38 MAPK-mediated MK2 phosphorylation.^18^ CMPD1 selectively inhibits MK2 phosphorylation with apparent *K*_i_ of 330 nM and does not inhibit p38 MAPK-mediated phosphorylation of other two substrates, MBP and ATF2.

In this study, we aimed to investigate whether MK2 inhibition could result in attenuation of glioblastoma cell proliferation and whether allosteric MK2 inhibitors, such as CMPD1, could be used as new leads for development of glioblastoma treatments. We demonstrate that while CMPD1 exhibited cytotoxic effects in glioblastoma cells, it did not inhibit MK2 at concentrations corresponding to the cytotoxic activity. In addition, at higher concentrations CMPD1 induced activation of the p38 MAPK–MK2 pathway. MK2 knockdown and two structurally unrelated and ATP-competitive MK2 inhibitors, MK2 inhibitor III^19^ and PF-3644022,^17^ were not cytotoxic to glioblastoma cells. Together, these data suggest that the cell death caused by CMPD1 is not mediated through the p38 MAPK–MK2 pathway. We identified CMPD1 as a novel microtubule-depolymerizing agent that induces mitotic arrest and promotes apoptosis. We also demonstrate that CMPD1 is less toxic to non-malignant cells when compared with clinically used tubulin inhibitors paclitaxel and vinblastine.

## Results

### CMPD1 attenuates glioblastoma cell viability, but does not inhibit MK2 phosphorylation

**Figure 1:**
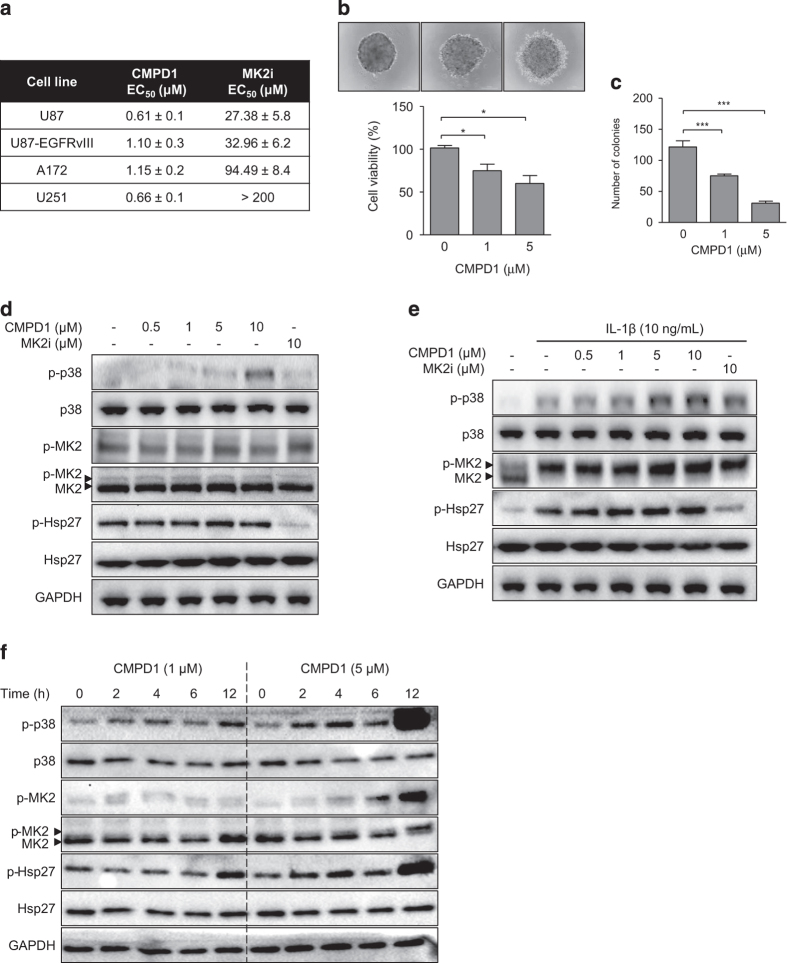
Cytotoxic efficacy of MK2 inhibitors in glioblastoma cells. (**a**) U87, U87-EGFRvIII, A172, and U251 cells were treated with CMPD1 or MK2i (0.001–200 *μ*M; 72 h) and AlamarBlue cell viability assay was performed. EC_50_ values were calculated by non-linear regression analysis. Data are expressed as mean±S.E.M. from at least three independent experiments. (**b** and **c**) U87 spheroids were treated with CMPD1 (72 h) and AlamarBlue cell viability assay was performed (**b**). In parallel, spheroids were dispersed and cells were allowed to form colonies for 12–15 days (**c**). Data are expressed as mean±S.E.M. (*n*=3; *P<0.05, ***P<0.001, one-way ANOVA followed by Newman–Keuls test). (**d**) U87 cells were incubated with CMPD1 or MK2i (2 h) and cell lysates were analysed with western blotting using indicated antibodies. (**e**) U87 cells were pre-treated with CMPD1 or MK2i (2 h) and stimulated with IL-1*β* (10 ng/ml) for 15 min. Cell lysates were analysed with western blotting using indicated antibodies. (**f**) U87 cells were treated with CMPD1 for indicated time and cell lysates analysed with western blotting using indicated antibodies. In (**d**–**f**), representative images of three independent experiments are shown.

To investigate the cytotoxic activity of MK2 inhibitors, we determined EC_50_ values for two structurally unrelated MK2 inhibitors in a cell viability assay. We used U87, U87-EGFRvIII, A172, and U251 glioblastoma cell lines, which possess different genetic backgrounds. U251 and A172 cells harbour p53 mutation, whereas U87 cells express wild-type p53. In addition, U87-EGFRvIII cells express constitutively active epidermal growth factor receptor variant III (EGFRvIII), a mutation found in 30% of glioblastomas and associated with poor patients survival.^20^ CMPD1 potently reduced viability of glioblastoma cells with EC_50_ ranging between 0.6 and 1.2 *μ*M (Figure 1a). On the other hand, MK2 inhibitor III (thereafter MK2i) exhibited weaker activity with EC_50_ of 27.4 *μ*M in U87 cells. Treatment of U251 cells with CMPD1 (200 *μ*M) caused only 30% reduction in cell viability. Due to the differences between these two MK2 inhibitors, we employed a third MK2 inhibitor PF-3644022. This compound exhibited EC_50_ of 52.2 *μ*M in U87 cells (Supplementary Figure S1A). Similarly, p38 MAPK inhibitor SB203580 (10 *μ*M) did not affect the viability of U87 glioblastoma cells (data not shown).

To further demonstrate the activity of CMPD1 in an assay closer mimicking the tumour *in vivo*, spheroids were derived from U87 cells and treated with CMPD1. Representative images and the quantitative bar graph demonstrate a decrease in cell viability (Figure 1b). Moreover, CMPD1 significantly suppressed the colony forming ability of U87 cells derived from treated spheroids (Figure 1c). CMPD1 also reduced viability of primary glioblastoma cells (EC_50_=1.55 *μ*M) as well as viability of astrocytoma, lung and colon cancer cell lines (Supplementary Figure S1B). This activity of CMPD1, however, could not be reproduced with other MK2 inhibitors MK2i and PF-3644022.

To address the differences in the activities of MK2 inhibitors, we analysed the phosphorylation of MK2 and its downstream substrate heat-shock protein 27 (Hsp27) in U87 cells treated with CMPD1 and MK2i (Figure 1d). CMPD1 is an inhibitor of MK2 activation and is expected to inhibit the phosphorylation of MK2 by p38 MAPK, whereas MK2i binds to the phosphorylated MK2 and blocks its activity to phosphorylate Hsp27.^19^ Surprisingly, cells treated with CMPD1 at cytotoxic concentrations (0.5–10 *μ*M, 2 h) did not show any changes in the phosphorylation of MK2 and Hsp27 (Figure 1d). On the other hand, MK2i at 10 *μ*M, which is below its EC_50_ value, effectively reduced basal Hsp27 phosphorylation in U87 cells (Figure 1d).

We further examined the efficacy of these two compounds in U87 cells stimulated with IL-1*β*, which is potent activator of the p38 MAPK–MK2–Hsp27 pathway (Figure 1e). In line with the previous data (Figure 1d), CMPD1 (0.5–10 *μ*M) did not inhibit the phosphorylation of MK2 and Hsp27, while MK2i (10 *μ*M) effectively reduced Hsp27 phosphorylation (Figure 1e). Intriguingly, CMPD1 at higher concentrations (5–10 *μ*M) increased phosphorylation of p38 MAPK in both unstimulated (Figure 1d) and IL-1*β* stimulated (Figure 1e) U87 cells. We therefore performed a thorough time- and dose-dependent analysis to determine the effect of CMPD1 on the p38 MAPK–MK2–Hsp27 axis in U87 cells (Figure 1f). Indeed, treatment of U87 cells with CMPD1 (1 and 5 *μ*M) resulted in a dose- and time-dependent phosphorylation of p38 MAPK, MK2, and Hsp27 (Figure 1f). Similar results were obtained after longer incubations (up to 72 h) with CMPD1 (Supplementary Figures S1C and D). As p38 MAPK is a stress response kinase, the activation of the p38 MAPK–MK2 pathway by CMPD1 indicated that the drug treatment could lead to cellular stress.

### CMPD1 induces cell-cycle arrest, accumulation of polyploidy and alters expression of G2/M regulatory proteins

**Figure 2:**
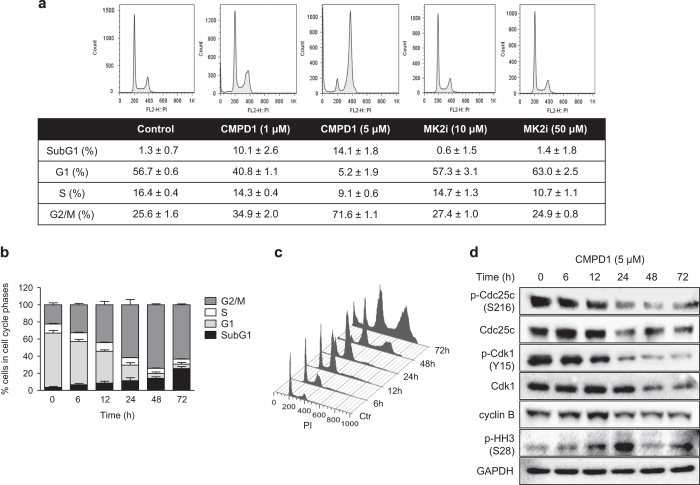
CMPD1 induces G2/M arrest in glioblastoma cells. (**a**) U87 cells were treated with CMPD1 or MK2i (48 h) and cell-cycle distribution was analysed by flow cytometry. Proportion of cells in each cell-cycle phase is expressed as mean±S.E.M. from three independent experiments; cells with DNA content >4n were excluded from the analysis. (**b**) U87 cells were treated with CMPD1 (5 *μ*M) for indicated time and cell-cycle distribution was analysed by flow cytometry. Bars represent proportion of cells in each cell-cycle phase; data are expressed as mean±S.E.M. from three independent experiments. (**c**) A representative FACS plot for U87 cells treated with CMPD1 (5 *μ*M) for indicated time demonstrating changes in 2n, 4n, and 8n populations. (**d**) U87 cells were treated with CMPD1 for indicated time and cell lysates analysed with western blotting using indicated antibodies. Representative images of three independent experiments are shown.

To understand the mechanism underlying the anti-proliferative activity of CMPD1 in glioblastoma cells, we studied the changes in the cell-cycle distribution after drug treatment. Treatment of U87 cells with CMPD1 (1 and 5 *μ*M, 48 h) resulted in dose-dependent G2/M arrest and appearance of SubG1 population (Figure 2a). In contrast, MK2i (10 and 50 *μ*M) did not affect the percentage of cells in G2/M phase and did not increase SubG1 proportion of U87 cells. We only observed 1.7% (10 *μ*M) and 5.7% (50 *μ*M) decrease in the S-phase population with MK2i, indicative of slower cell proliferation (Figure 2a). Comparable reduction in the percentage of cells in the S-phase was observed with the p38 MAPK inhibitor SB203580 (Supplementary Figure S2A). Further analysis revealed that CMPD1 (5 *μ*M) caused a time-dependent increase in G2/M arrest starting as early as 6 h after treatment, with 60% of the cells being arrested by 24 h (Figures 2b and c). A gradual increase in SubG1 phase, corresponding to apoptotic cells, reached 25% by 72 h (Figure 2b). CMPD1 also induced accumulation of a polyploid cell population (Figure 2c; Supplementary Figure S2B).

To investigate this further, we examined the expression and phosphorylation of the proteins regulating G2/M, such as cyclin-dependent kinase 1 (Cdk1) and cell division cycle phosphatase 25c (Cdc25c). CMPD1 (5 *μ*M) caused a time-dependent decrease in the phosphorylation and expression of Cdk1 and Cdc25c (Figure 2d). The decrease in the inhibitory phosphorylation of Cdc25c (S216) activates this phosphatase, which in turn leads to dephosphorylation (thus activation) of Cdk1 (Figure 2d), resulting into the progression to mitosis. In support, CMPD1 upregulated cyclin B expression 12 h upon drug treatment, accompanied with a gradual increase in the expression of the mitotic marker p-HH3 (S28). The subsequent decrease in cyclin B and p-HH3 levels after 24 h indicates mitotic exit. Treatment of U87 cells with CMPD1 (1 *μ*M) resulted in comparable results (Supplementary Figures S2C and D). Taken together, the interplay between these G2/M regulators underpins the changes in the cell-cycle progression induced by CMPD1.

### CMPD1 triggers apoptosis in U87 cells and regulates expression of anti-apoptotic proteins

**Figure 3:**
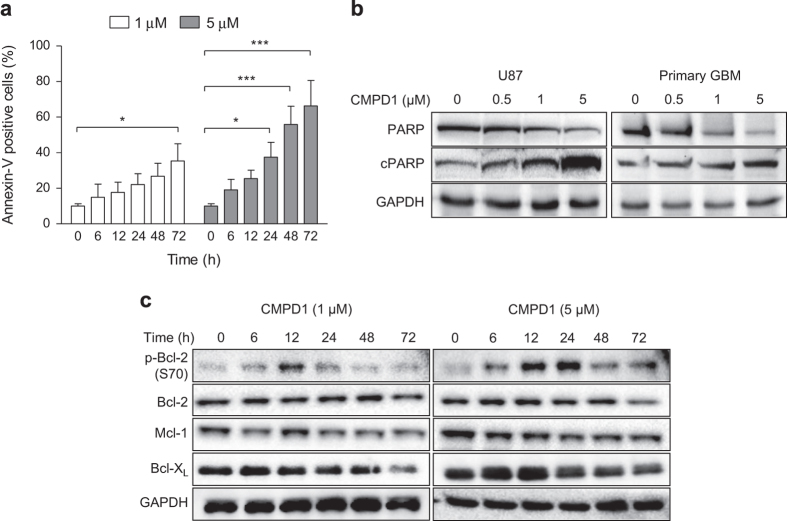
CMPD1 induces apoptosis and changes expression of Bcl-2 family proteins. (**a**) U87 cells were treated with CMPD1 for indicated time and the percentage of Annexin-V-positive cells was determined by flow cytometry. Data are expressed as mean±S.E.M. (*n*=3; *P<0.05, ***P<0.001, two-way ANOVA followed by Bonferroni post-test). (**b**) U87 and primary human glioblastoma cells were treated with CMPD1 (48 h) and analysed for expression of PARP (116 kDa) and cleaved PARP (85 kDa) using western blotting. Representative images of three independent experiments are shown. (**c**) U87 cells were treated with CMPD1 for indicated time and cell lysates analysed with western blotting using indicated antibodies. Representative images of three independent experiments are shown.

To investigate whether apoptosis underlies the decrease in viability of cells treated with CMPD1 (Figure 1a), we analysed apoptosis in U87 cells by Annexin-V staining and immunoblotting of the cleaved poly-(ADP-ribose)-polymerase (cPARP). CMPD1 (1 and 5 *μ*M, 72 h) increased the amount of apoptotic cells to 38 and 56%, respectively (Figure 3a). In contrast, MK2i or SB203580 did not induce apoptosis in U87 cells (Supplementary Figures S3A and B). Consistent with an apoptotic mechanism for cell death, we observed a significant increase in the appearance of cleaved PARP after 48 h of cell treatment with CMPD1 (1 and 5 *μ*M, Figure 3b).

As CMPD1 induced mitotic arrest (Figure 2) and apoptosis (Figures 3a and b) in U87 cells, we investigated the effect of this inhibitor on the expression of anti-apoptotic Bcl-2 proteins that link the mitotic arrest to the induction of apoptosis.^21^ Treatment of U87 cells with CMPD1 resulted in phosphorylation of Bcl-2 and downregulation of Mcl-1 and Bcl-X_L_ (Figure 3c). We further show that the Mcl-1 downregulation in CMPD1-treated U87 cells is due to its proteosomal degradation, the proteasome inhibitor MG132 reversed the CMPD1-induced Mcl-1 degradation (Supplementary Figure S3C). As phosphorylation of Bcl-2 and degradation of Mcl-1 and Bcl-X_L_ allow activation of the apoptotic proteins Bax and Bak,^22^ these data could explain the onset and extent of apoptosis in cells treated with CMPD1.

### Cell death caused by CMPD1 is independent of MK2

**Figure 4:**
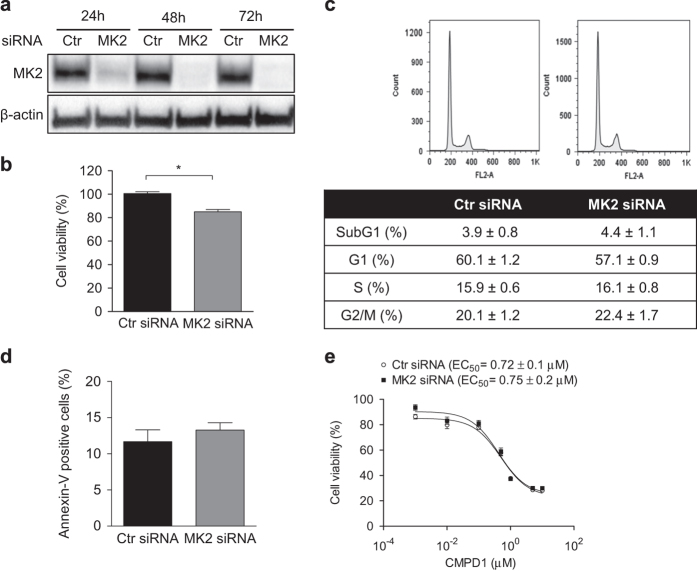
Effect of MK2 knock-down on viability, cell cycle, and apoptosis in U87 cells. (**a**) U87 cells were reverse-transfected with control or MK2-targeting siRNA (5 nM). Cells were harvested at indicated time and MK2 expression determined with western blotting. Representative images of three independent experiments are shown. (**b**) Cells were transfected as in (**a**) and AlamarBlue cell viability assay was performed 72 h post transfection. Data are expressed as mean±S.E.M. (*n*=5; *P<0.05, unpaired *t*-test). (**c** and **d**) Cells were transfected as in (**a**), and cell-cycle analysis (**c**) and Annexin-V staining (**d**) were performed 48 h post transfection. Data are expressed as mean±S.E.M. (*n*=3). (**e**) U87 cells were transfected as in (**a**) overnight, treated with CMPD1 (72 h) and AlamarBlue cell viability was performed. EC_50_ was calculated using Prism 5 GraphPad Software. Data are expressed as mean±S.E.M. (*n*=4).

Treatment of U87 with the non-ATP competitive MK2 inhibitor CMPD1 affected the cell-cycle progression and induced apoptosis (Figures 2 and 3); however, at doses corresponding to the apoptotic activity, CMPD1 did not prevent activation of the MK2 pathway (Figures 1d and e). MK2i, on the other hand, potently inhibited MK2-dependent phosphorylation of Hsp27 (Figures 1d and e), but did not demonstrate any cytotoxic activity (Figure 1a and Supplementary Figure S3A). We therefore aimed to investigate whether MK2 is the target of CMPD1 responsible for the cell death. First, we assessed whether genetic knock-down of MK2 in U87 could reproduce results obtained with CMPD1. Despite >90% knockdown efficiency (Figure 4a), siRNA-mediated deletion of MK2 caused only 15% decrease in the viability of U87 cells (Figure 4b). Similar results were obtained in U251 and U87-EGFRvIII glioblastoma cells (Supplementary Figures S4A and B). This result coincided with the decrease in cell viability upon MK2i treatment (10 *μ*M caused 18% decrease in the cell viability, data not shown). Furthermore, MK2 knockdown in U87 cells did not change the cell-cycle distribution (Figure 4c), did not induce apoptotic cell death (Figure 4d) and did not affect U87 cell morphology (Supplementary Figure S4C). Finally, MK2 knockdown did not alter the EC_50_ value of CMPD1 in the cell viability assay (Figure 4e), implicating that MK2 is dispensable for CMPD1 toxicity in U87 cells.

### CMPD1 inhibits tubulin polymerization and induces mitotic spindle defects

Our data suggested that CMPD1 is a mitotic inhibitor and a pro-apoptotic agent that displays its activity independently of MK2. This together with the observation that CMPD1 caused pronounced morphological changes in the appearance of U87 cells early upon drug treatment (Supplementary Figure S5A) prompted us to hypothesize that CMPD1 might be targeting the cytoskeleton. In support, we observed similar changes in U87 morphology when cells were treated with the microtubule depolymerizing agent nocodazole (data not shown), and both CMPD1 (Figure 1f) and nocodazole (Supplementary Figure S5B) activated the p38 MAPK–MK2–Hsp27 pathway.

**Figure 5:**
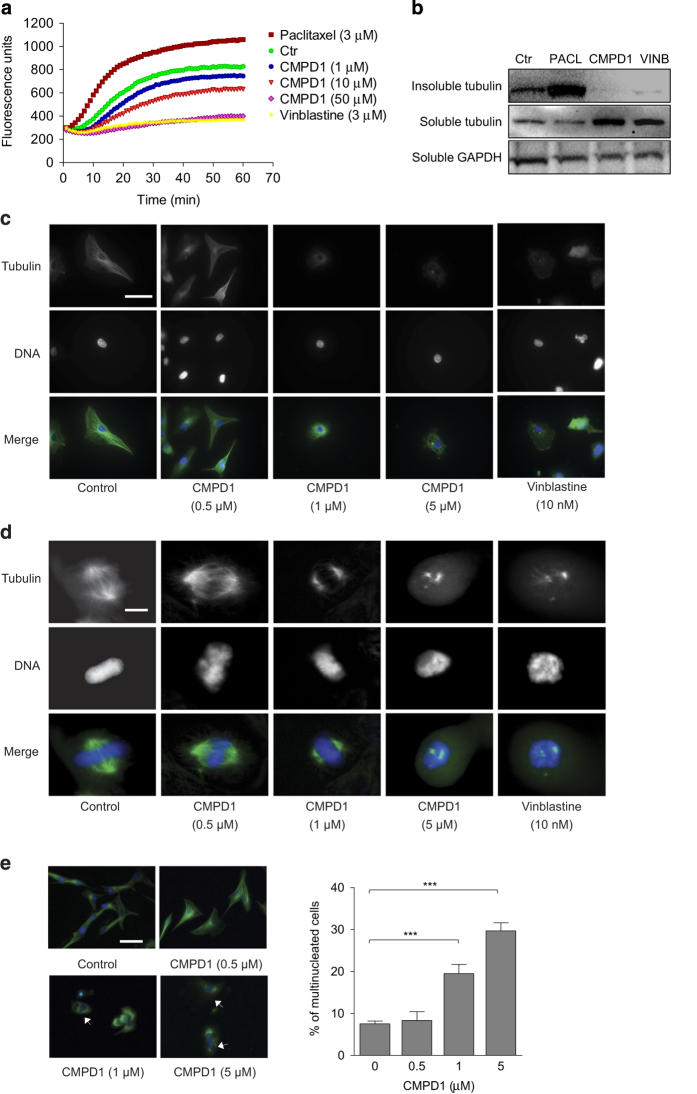
CMPD1 exhibits a mechanism of action consistent with microtubule disruption. (**a**) CMPD1 inhibits polymerization of tubulin *in vitro*. Porcine brain tubulin was incubated with paclitaxel, vinblastine, or CMPD1 and assembly of microtubules was monitored by an increase in fluorescence. Data are expressed as mean from at least three independent experiments; each data point was performed in triplicates. (**b**) *In vivo* tubulin polymerization was determined in U87 cells treated with paclitaxel (300 nM), CMPD1 (5 *μ*M), or vinblastine (300 nM) for 1 h. Cell lysates were centrifuged to separate the insoluble and soluble tubulin, and separated fractions were analysed with western blotting. Insoluble tubulin represents the polymerized fraction and soluble tubulin represents the unpolymerized fraction. GAPDH served as a loading control. Representative blots from three independent experiments are shown. (**c**) U87 cells treated with CMPD1 or vinblastine (24 h) were stained with Alexa488-labelled anti-*β*-tubulin antibody (green) or DAPI (blue). CMPD1 treatment profoundly disturbed the microtubule network. White scale bar indicates 50 *μ*m. Representative images of three independent experiments are shown. (**d**) U87 cells were treated with CMPD1 or vinblastine (48 h) and tubulin and DNA stained as in (**c**). CMPD1 treatment caused defective mitotic spindle formation, similarly to the effect of vinblastine. Representative images from three independent experiments are shown. White scale bar indicates 2 *μ*m. (**e**) U87 cells were treated with CMPD1 (48 h) and tubulin and DNA stained as in (**d**). Multinucleated cells were manually counted and at least 200 cells were counted for each sample. Representative images of three independent experiments are shown, arrows indicate multinucleated cells. White scale bar represents 50 *μ*m. Data in the bar graph are expressed as mean±S.E.M. (*n*=3; ***P<0.001, one-way ANOVA followed by Newman–Keuls post-test)

Indeed, CMPD1 inhibited tubulin polymerization *in vitro* in a dose-dependent manner and the effect was similar to the effect induced by the microtubule-destabilizing agent vinblastine (Figure 5a). Paclitaxel and vinblastine induced a marked increase and decrease in tubulin polymerization, respectively. The tubulin-targeting activity of CMPD1 was confirmed in a cell-based polymerization assay using 5 *μ*M CMPD1 (Figure 5b). In line with the published data,^23^ paclitaxel increased the insoluble polymerized tubulin fraction, whereas CMPD1 and vinblastine reduced the polymerized fraction compared with untreated U87 cells (Figure 5b).

Next, the effect of CMPD1 on the arrangement and distribution of the microtubule network in U87 cells was studied *in situ* by immunofluorescence. In non-mitotic cells, microtubules radiate from the microtubule-organizing centre located at the centrosome in the cytoplasm maintaining cell shape. The treatment of U87 cells with CMPD1 disrupted the microtubule cytoskeleton similar to vinblastine, leading to a loss of microtubules and long microtubule fibres could rarely be observed in these cells (Figure 5c). The consequence of microtubule depolymerization induced by CMPD1 was disrupted spindle assembly (Figure 5d). In untreated U87 cells undergoing mitosis, cellular microtubules are normally assembled into bipolar mitotic spindles to guarantee equal segregation of the chromosomes (Figure 5d, control images). However, treatment with CMPD1 resulted into defective spindle formation and chromosome misalignment similar to vinblastine (Figure 5d). Finally, immunofluorescence analysis of U87 cells treated with CMPD1 (1 and 5 *μ*M, 48 h) revealed an increase in multi-nucleation (Figure 5e), indicating failed mitosis. Collectively, these data indicate that CMPD1 interferes with mitosis progression by disrupting mitotic spindle assembly.

### CMPD1 is selectively toxic to glioblastoma cells

**Figure 6:**
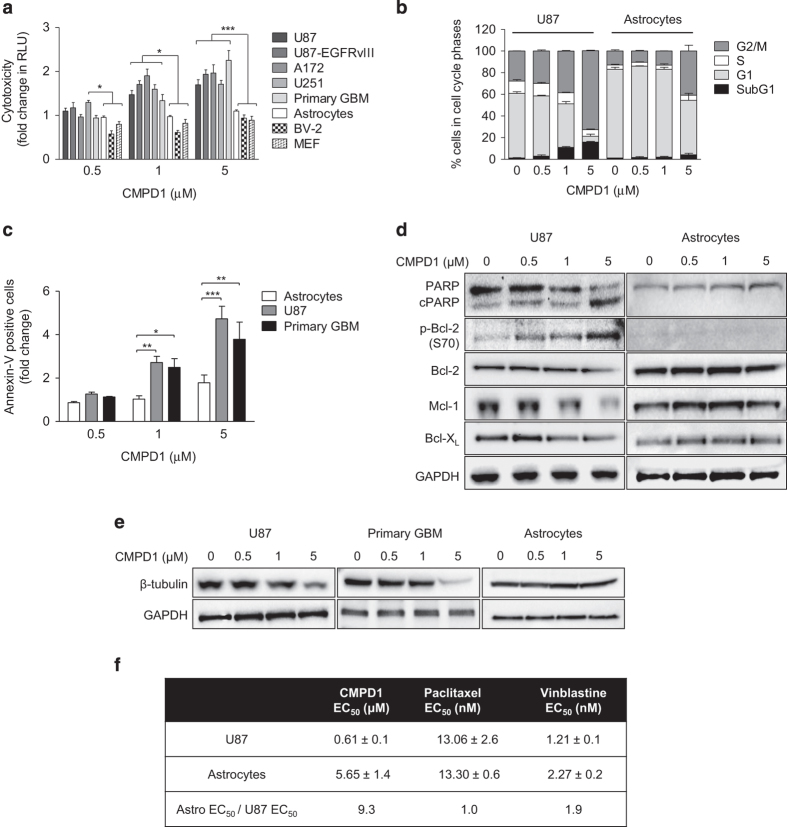
CMPD1 is selectively toxic to glioblastoma cells. (**a**) Glioblastoma cell lines (U87, U87-EGFRvIII, A172, and U251), primary human glioblastoma, human astrocytes, mouse microglial BV-2 cell line, and MEF cells were incubated with CMPD1 (72 h). Cytotoxicity was determined by luminescence quantification of adenylate kinase in cell supernatants (ToxiLight bioassay). Relative light unit (RLU) values were normalized against untreated cells. Data are expressed as mean±S.E.M. (*n*=3; *P<0.05, ***P<0.001, two-way ANOVA followed by Bonferroni post-test). (**b**) U87 and primary human astrocytes were treated with CMPD1 (48 h) and cell-cycle distribution was determined by flow cytometry. Data are expressed as mean±S.E.M. (*n*=3). (**c**) U87, primary human glioblastoma, and astrocytes were treated with CMPD1 (48 h) and apoptosis determined by Annexin-V staining. The percentage of Annexin-V-positive cells was normalized against untreated cells. Data are expressed as mean±S.E.M. (*n*=3; *P<0.05, **P<0.01, ***P<0.001, two-way ANOVA followed by Bonferroni post-test). (**d**) U87 and primary human astrocytes were treated with CMPD1 (48 h) and cell lysates analysed by western blotting using indicated antibodies. Representative blots from three independent experiments are shown. (**e**) U87 cells, primary human glioblastoma and astrocytes were incubated with CMPD1 (48 h) and effect on tubulin expression was determined by western blotting. (**f**) U87 cells and primary human astrocytes were treated with CMPD1, paclitaxel or vinblastine (0.001–10 *μ*M; 72 h) and AlamarBlue cell viability assay was performed. EC_50_ values were calculated by non-linear regression analysis. Data are expressed as mean±S.E.M. (*n*=11 for CMPD1; *n*=3 for paclitaxel and vinblastine).

As CMPD1 displayed apoptotic activity in glioblastoma cells, we investigated whether this compound is also cytotoxic to the non-malignant cells. The cytotoxicity of CMPD1 was assessed with the ToxiLight assay using a panel of non-malignant and glioblastoma cell lines. No toxic effect was observed in assays employing primary human astrocytes, microglial BV-2 cell line and mouse embryonic fibroblasts (MEFs), while CMPD1 was cytotoxic to glioblastoma cells (Figure 6a).

To further assess the different sensitivity of non-malignant cells to CMPD1, we compared the cell-cycle progression of astrocytes and U87 cells treated with CMPD1 (Figure 6b). In astrocytes, CMPD1 (5 *μ*M) promoted G2/M arrest (27.8% increase relative to control), which was significantly lower than G2/M arrest in U87 cells treated with CMPD1 (45.3% increase relative to control). Furthermore, there was minimal increase in the astrocytes SubG1 fraction, in contrast to CMPD1-treated U87 cells (Figure 6b; Supplementary Figure S6A). In addition, CMPD1 caused a dose-dependent increase of polyploidy in U87 cells; however, astrocytes were resistant to polyploidy induction (Supplementary Figure S6B).

We next compared the extent of apoptosis upon treatment of astrocytes, U87 and primary glioblastoma cells with CMPD1 for 48 h (Figure 6c). Consistently with previous findings, the percentage of apoptotic cells was 2- to 3-fold higher in U87 and primary glioblastoma cells treated with CMPD1 (1 and 5 *μ*M) when compared with astrocytes treated with CMPD1. Furthermore, immunoblotting analysis of astrocytes and U87 cells cultured with CMPD1 demonstrated an increase in cleaved PARP expression in U87 cells, but not in astrocytes (Figure 6d).

To better understand the increased sensitivity of glioblastoma cells to CMPD1 when compared with primary astrocytes, we also investigated the changes in the expression of anti-apoptotic proteins in both cell types (Figure 6d). In line with the previous findings (Figure 3c), CMPD1 induced phosphorylation of Bcl-2 and downregulation of Mcl-1 and Bcl-X_L_ in U87 cells (Figure 6d). However, no changes in the phosphorylation and expression of these proteins were observed in astrocytes treated with CMPD1, which could explain minimal apoptotic activity of CMPD1 in non-malignant astrocytes. In addition to these results, the characteristic changes in U87 morphology upon treatment with CMPD1 were not evident in astrocytes (Supplementary Figure S6C). Furthermore, we observed a marked reduction in tubulin levels in U87 and primary glioblastoma cells treated with CMPD1 (48 h), but not in astrocytes (Figure 6e), suggesting that CMPD1 has less pronounced effect on tubulin cytoskeleton in astrocytes.

Finally, we compared activity and selectivity of CMPD1 to the clinically used tubulin inhibitors vinblastine and paclitaxel. In the viability assays using U87 cells, paclitaxel and vinblastine demonstrated nanomolar efficacy (EC_50_=13.1 and 1.21 nM, respectively; Figure 6f), whereas CMPD1 was less potent (EC_50_=0.61 *μ*M). However, while paclitaxel and vinblastine were equally cytotoxic to astrocytes (EC_50_=13.3 and 2.27 nM, respectively), CMPD1 attenuated the viability of astrocytes with EC_50_ value of 5.65 *μ*M, which is in agreement with the cytostatic effect and G2/M arrest induced by 5 *μ*M CMPD1 (Figure 6b). In calculating the ratio of EC_50_ (astrocytes) to EC_50_ (U87), CMPD1 was 9.3 times more efficacious in U87 cells compared with astrocytes. The efficacy ratio for paclitaxel and vinblastine was 1.0 and 1.9, respectively (Figure 6f).

## Discussion

The p38 MAPK–MK2 signalling has been identified as one of the cell-cycle checkpoint regulators alongside Chk1/2 and Wee1 pathways.^24^ Inhibition of Chk1/2 or Wee1 is cytotoxic to various cancer cells including glioblastoma, and resulted in increased synthetic lethality when combined with DNA damaging agents.^7–9,25,26^ Similarly, MK2 has been proposed as a chemo-sensitizing target in cancers that express wild-type p53 or are p53 deficient.^13–15,27^ We have shown that activated MK2 is overexpressed in tumours prior therapy, particularly in tumours with mutated p53,^28^ suggesting a potential role for MK2 in cancer independently of the p53 functionality and chemotherapy-induced DNA damage. We used the non-ATP competitive inhibitor of MK2 activation, CMPD1, together with the genetic MK2 knock-down and two ATP-competitive MK2 inhibitors MK2i and PF-3644022 to address the question whether MK2 inhibition could be a useful strategy to attenuate growth of glioblastoma tumours.

We find that in the absence of DNA damaging agents, MK2 inhibition does not induce cell death in glioblastoma cells. MK2 inhibitors MK2i and PF-3644022 were not effective in reducing viability of glioblastoma cells, despite on-target inhibition in the cells. In support, genetic downregulation of MK2 did not activate apoptosis or changed the cell cycle of glioblastoma cells. Thus, in the absence of DNA damaging agents, MK2 appears to be dispensable for the survival of cancer cells. This finding together with the apparently normal phenotype of MK2 knock-out mice^29^ suggests that MK2 inhibition can be compensated by other genes. Intriguingly, MK2 activity is crucial for survival of cancer cell when exposed to DNA damaging chemotherapy.^13,27^

Furthermore, this study presents the important findings that CMPD1 attenuates viability of cancer cells, but its activity is independent of MK2. CMPD1 (*K*_i_=330 nM) prevents p38 MAPK-mediated phosphorylation of MK2 *in vitro*.^18^ Several cell-based studies employed CMPD1 as a MK2 inhibitor at various concentrations (0.3–50 *μ*M^30–34^); but only one study demonstrated that CMPD1 at 10 *μ*M inhibited TNF-*α* induced activation of the MK2 pathway.^35^ We present here that in U87 glioblastoma cells, CMPD1 at concentrations up to 10 *μ*M did not inhibit the activation of MK2 and Hsp27. We further demonstrate that CMPD1 at concentrations far below 10 *μ*M inhibits tubulin polymerization in glioblastoma cells, which induces cellular stress and consequent late activation of the p38 MAPK–MK2 pathway. We propose that CMPD1 is primarily a microtubule-targeting agent selectively inhibiting tubulin polymerization in glioblastoma cells, causing mitotic spindle defects and apoptotic cell death. In light of these findings, the use of CMPD1 as a MK2 inhibitor in cells and in *in vivo* models should be carefully considered.

It is commonly assumed that additional targets of kinase inhibitors will be other kinases. However, tubulin is emerging as one of the most common off-targets for kinase inhibitors. Tubulin-targeting activity has been reported for kinase inhibitors tivantinib and IC261.^36–38^ These compounds were developed as inhibitors of c-Met and CK1, respectively; but further studies demonstrated that their cytotoxic activity is due to inhibition of tubulin polymerization. Intriguingly, these effects were not reported by the pharmaceutical companies that developed these compounds, but by academic groups. Furthermore, in the case of tivantinib this happened after the drug candidate had entered numerous Phase II/Phase III clinical trials as a c-Met inhibitor. To our best knowledge, only Bristol-Myers Squibb reported that the cytotoxicity of LIMK1 inhibitors developed in their pipeline is mediated by tubulin rather than the targeted kinase.^39^ Recently, increasing number of studies report the development of inhibitors with dual activity against kinases and microtubules.^40,41^

Whether the drug candidate is a molecularly-targeted agent (e.g., kinase inhibitor), chemotherapeutic drug (e.g., microtubule-targeting agent), or both will affect basic research as well as drug development in several ways and will have profound implications on selection of patients in clinical trials. Thus, the precise understanding of the mechanism of action is crucial for high-quality and reproducible results; although it is not always trivial to recognize that the intended target does not mediate the observed effect of a compound. This is especially challenging with oncology agents where viability screens are the primary drug discovery platforms. In addition, inhibitors targeting checkpoint or mitotic kinases induce mitotic arrest, which is also the mechanism of action for microtubule-targeting agents. Furthermore, several studies demonstrate that mismatching phenotypes after genetic and pharmacological inhibition do not necessarily imply off-target activity. The non-catalytical functions of kinases, such as scaffolding or transporter roles, have been discovered based on differences in assays using a selective kinase inhibitor (inhibiting only the catalytic activity) and genetic knock-down (inhibiting catalytic as well as other activities^42–44^). Thus, complex mechanistic studies are necessary to correctly identify the target(s). In our study, the changes in cell morphology as early as 1 h of CMPD1 treatment that are not typically seen when cells are treated with *bona fide* kinase inhibitors indicated that MK2 might not be the molecular target of CMPD1. Taking into account potential non-kinase functions of MK2, we included two additional MK2 inhibitors MK2i and PF-3644022. Because the phenotype obtained with these inhibitors corresponded to the phenotype observed after MK2 knock-down, we recognized that the CMPD1 activity is not mediated by MK2.

Taken together, this is the first study to show that the CMPD1 exhibits apoptotic activity in cancer cells. This activity is independent of MK2 and we identified CMPD1 as a novel tubulin-depolymerizing agent. This study together with other reports of tubulin-targeting kinase inhibitors indicate that there could be a structural similarity between kinases and tubulin isoforms. Better understanding of the pharmacophore that defines affinity to both kinases and tubulin isoforms would be a powerful tool to predict the off-target activity in advance. Until this has been achieved, however, potential tubulin-targeting activity should be assessed in the early stages of kinase inhibitors discovery campaigns. Finally, detailed preclinical pharmacology studies should be performed and disclosed, to develop drug candidates or experimental tools with well-defined mechanism of action.

## Materials and methods

### Cell culture and reagents

U87 and U251 glioblastoma cells were purchased from European Collection of Cell Cultures (ECACC, Salisbury, UK). A172 and U138 glioblastoma, SW-1088 astrocytoma, lung NCI-H23, A549 and colon HCT116, murine microglial BV-2 cells and MEFs were purchased from American Type Culture Collection (ATCC, Manassas, VA, USA). U87-EGFRvIII glioblastoma cells were provided by their lab of origin^45^ and tested for EGFR mutation and stable expression by western blotting. Primary glioblastoma cells were isolated from a tumour sample collected from a female patient who underwent surgery at the Centre for Minimally Invasive Neurosurgery in Sydney, Australia.^46^ Primary human astrocytes were isolated as described,^47^ the protocol has been approved by the Human Ethics Committee of The University of Sydney (HREC 2013/131) and Macquarie University (HEC 5201200411). U87, U87-EGFRvIII, U251, A172, U138, LN-18, BV-2, and A549 cell lines were cultured in DMEM (Life Technologies, Carlsbad, CA, USA) supplemented with 10% FBS (Sigma-Aldrich, St. Louis, MO, USA). Primary glioblastoma cells, astrocytes and NCI-H23, HCT116, and MEF cell lines were cultured in RPMI-1640 (Life Technologies) supplemented with 10% FBS (Sigma-Aldrich). The cumulative culture length of all cell lines was less than 2 months. Inhibitors CMPD1 (sc-203138) and MK2 inhibitor III (sc-221948) were from Santa Cruz Biotechnology (Santa Cruz, CA, USA). SB203580, PF-3644022, nocodazole, paclitaxel, vinblastine, and MG132 were purchased from Sigma-Aldrich.

### Cell viability and cytotoxicity assays

Cells (2×10^3^) were treated with vehicle or test compounds for 72 h. AlamarBlue reagent (Life Technologies) was added to the cell culture and fluorescence was measured with FLUOstar Omega microplate reader (BMG Labtech, Ortenberg, Germany) at 585 nm. EC_50_ values were determined by non-linear regression model using Prism 5 (GraphPad Software, La Jolla, CA, USA). Cytotoxicity was determined by quantitative analysis of adenylate kinase in the cell culture medium using the ToxiLight bioassay kit (Lonza, Walkersville, MD, USA) following the manufacturer’s instructions.

### Western blotting analysis

Cell lysates were separated with SDS-PAGE as described previously.^28^ Proteins were detected using specific primary antibodies against p38 MAPK (#9212), phospho-p38 MAPK (Thr180/Tyr182; #9215), MK2 (#3042), Hsp27 (#2402), phospho-Hsp27 (Ser82; #2401), Cdk1 (#9116), phospho-Cdk1 (Tyr15; #9111), phospho-Histone H3 (Ser28; #9713), cyclin B1 (#4135), Cdc25c (#4688), phospho-Cdc25c (Ser216; #4901), PARP (#95425), Bcl-2 (#2870), phospho-Bcl-2 (Ser70; #2870), Bcl-X_L_ (#2764), and Mcl-1 (#5453, all from Cell Signaling Technology, Danvers, MA, USA). Antibodies against phospho-MK2 (Thr334; #ab51018) and *β*-tubulin (#ab11308) were from Abcam (Cambridge, UK). Antibodies against *β*-actin (#A5316) and GAPDH (#737179) were from Sigma-Aldrich and Santa Cruz Biotechnology, respectively. Secondary antibodies were from Cell Signaling Technology. Detection was performed using the Immobilion Western HRP Substrate Luminol-Peroxidase reagent (MerckMillipore, Billerica, MA, USA) and the ChemiDoc MP System (Bio-Rad, Hercules, CA, USA). Band intensities were quantified by Image-Lab (Bio-Rad).

### siRNA transfection

siRNA transfection was performed using RNAiMax, validated MK2 Silencer Select siRNA (sense 5′-CAGUAUCUGCAUUCAAUCAATT-3′; #4390824) and negative control siRNA (#4390846) purchased from Life Technologies. Cells were reverse-transfected with 5 nM MK2 or control siRNA and incubated overnight. Next day, transfected cells were employed in assays as described.

### Flow cytometry

U87 cells or astrocytes (1–3×10^5^) were cultured in the presence of inhibitors for 6–72 h and fixed with 70% ice-cold ethanol overnight at 4 °C. To determine DNA content, cells were stained with 50 *μ*g/ml propidium iodide in the presence of 100 *μ*g/ml RNase A for 1 h. Samples were analysed using a FACSCalibur flow cytometer (BD Biosciences, San Jose, CA, USA) and FlowJo software (Tree Star Inc., Ashland, OR, USA).

### Apoptosis assay

U87 cells or astrocytes (1–3×10^5^) were treated with inhibitors for 6–72 h. After incubation, cells were stained with fluorescein isothiocyanate-labelled Annexin V and 5 *μ*g/ml 7-AAD for 20 min. Samples were analysed using Muse Cell analyzer and MuseSoft 1.2 software (MerckMillipore).

### Immunofluorescence microscopy

U87 cells (3×10^3^) were treated with test compounds for 24–48 h. Cells were fixed with ice-cold 100% methanol for 20 min at RT and blocked in 1% bovine serum albumin/PBS for 20 min. Cells were then incubated with *β*-tubulin antibody and Alexa488-conjugated anti-mouse IgG. Cell nuclei were counterstained with Prolong Gold mounting media with DAPI (4’,6’-diamidino-2-phenylindole; Life Technologies). Fluorescence images were captured under an Olympus IX81 inverted fluorescence microscope and analysed using AutoDeblur v 9.3 (AutoQuant Imaging, AutoQuant X, BioImaging Solutions, San Diego, CA, USA).

### *In vitro* tubulin polymerization assay

Fluorescence-based tubulin polymerization assay was conducted in final volume of 55 *μ*l using the Tubulin Polymerisation Assay kit (Cytoskeleton, Denver, CO, USA) as per the manufacturer’s instructions. Porcine brain tubulin was incubated with test compounds at 37 °C and fluorescence was measured using FLUOstar Omega microplate reader (BMG Labtech, excitation at 355 nm and emission at 460 nm).

### Cell-based tubulin polymerization assay

Cell-based tubulin polymerization assay was carried out as described.^23^ Briefly, U87 cells (4×10^5^) were treated with paclitaxel, vinblastine, or CMPD1 for 1 h and cells were lysed using hypotonic buffer (1 mM MgCl_2_, 2 mM EGTA, 0.5% Nonidet P-40, 20 mM Tris-HCl pH 6.8, 2 mM phenylmethylsulfonyl fluoride, 1% protease inhibitor cocktail). Soluble (unpolymerized) tubulin was separated from insoluble (polymerized) tubulin by centrifugation (14 300×*g* for 10 min) and analysed by western blotting.

### 3D spheroid culture and clonogenic assay

U87 (7×10^3^) cells were seeded in ultra-low attachment 96-well plates (Sigma-Aldrich) and left for 2 days to form spheroids (diameter 250 *μ*m). Spheroids were treated with CMPD1 for 72 h and cell viability was determined by AlamarBlue assay (as described above). Alternatively, spheroids were dispersed using Accumax (Millipore), cells (4×10^3^) were seeded into 10 cm Petri dishes and allowed to form colonies for 12–15 days. Colonies (50 cells=1 colony) were counted using ImageJ sofware (NIH).

### Statistical analysis

All results are mean±S.E.M. from at least three independent experiments. EC_50_ values were calculated by non-linear regression analysis using GraphPad Prism 5 software (GraphPad, La Jolla, CA, USA). An analysis of variance (ANOVA) or unpaired Student's *t*-test was performed with GraphPad Prism 5 software. *P*<0.05 was considered as significant.

## Abbreviations

ATF2: activating transcription factor 2
Bak: Bcl-2 antagonist/killer-1
Bax: Bcl-2-associated X protein
Bcl-2: B-cell lymphoma 2
Bcl-X_L_: B-cell lymphoma-extra large
Cdc25: cell division cycle phosphatase
Cdk: cyclin-dependent kinase
Chk1/2: checkpoint kinases 1 and 2
Hsp27: heat shock protein 27
IL: interleukin
MAPK: mitogen-activated protein kinase
MBP: myelin basic protein
Mcl-1: myeloid cell leukaemia 1
MEF: mouse embryonic fibroblast
MK2: MAPK-activated protein kinase 2
PARP: Poly-(ADP-ribose)-polymerase
*si*RNA: small-interfering RNA.
