# Supplementary material for: Cytotoxic activity of the MK2 inhibitor CMPD1 in glioblastoma cells is independent of MK2
Source: Cell Death Discov. 2015 Sep 7;1:15028–. doi: 10.1038/cddiscovery.2015.28 (PMC4979411; doi:10.1038/cddiscovery.2015.28)
Supplement: Supplementary Figures [file cddiscovery201528-s1.pdf]

Supplementary Figure S1

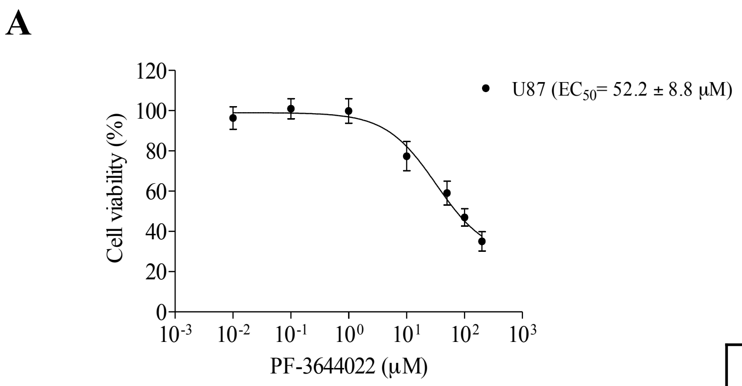

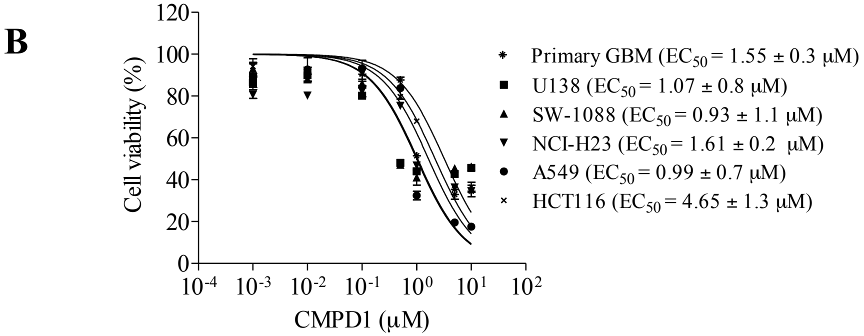

**Cell line**	**U138**	**SW-1088**	**NCI-H23**	**A549**	**HCT 116**
Tumor type	Glioblastoma	Astrocytoma III	Lung	Lung	Colon

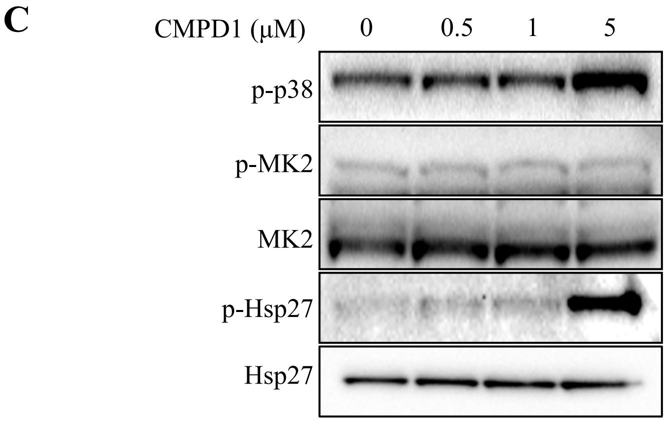

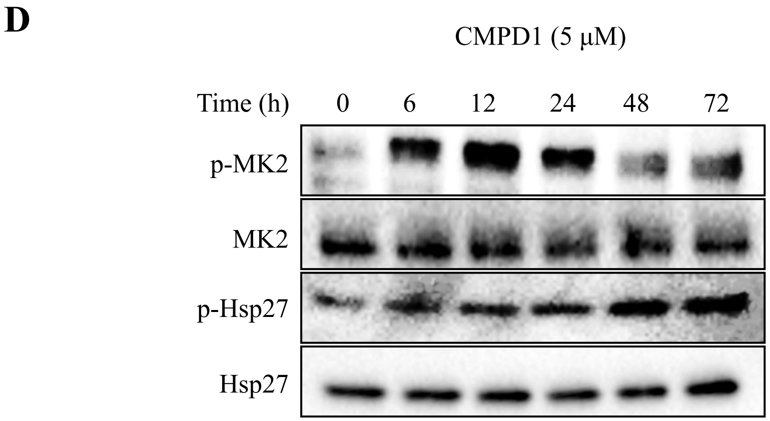

Supplementary Figure S1. (A) U87 cells were treated with PF-3644022 (72 h) and AlamarBlue cell viability assay was performed. Data are expressed as mean ± SEM (n = 3). **(B)** Cancer cells were treated with CMPD1 (72 h) and AlamarBlue cell viability was performed. Data are expressed as mean ± SEM (n = 2). **(C-D)** U87 cells were incubated with CMPD1 for **(C)** 48 h or **(D)** 6-72 h and cell lysates were analysed by Western blotting with indicated antibodies. Representative blots of three independent experiments are shown.

Supplementary Figure S2

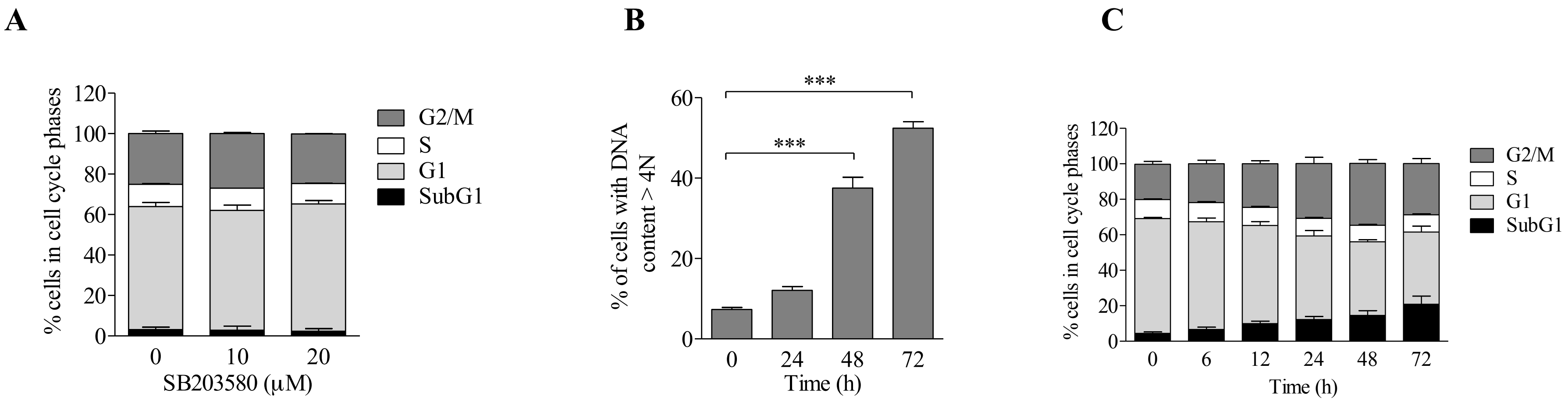

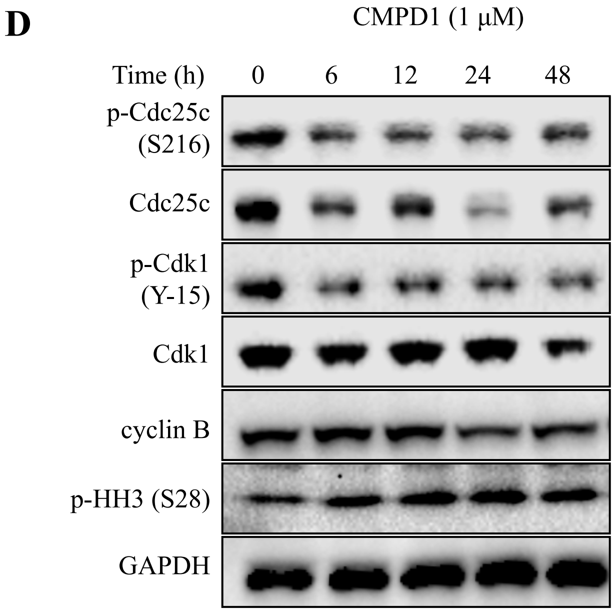

Supplementary Figure S2. (A) U87 cells were treated with SB203580 (48 h) and cell cycle distribution was analysed by flow cytometry. Data are expressed as the mean ± SEM (n=3). **(B)** U87 cells were treated with CMPD1 (5 μM) for indicated time and percentage of cells with DNA content greater than 4n was determined by flow cytometry. Data are expressed as the mean ± SEM (n = 3; ***P < 0.001, 1-way ANOVA followed by Newman-Keuls post-test). **(C)** U87 cells were treated with CMPD1 (1 μM) for indicated time and cell cycle distribution analysed as in (A). Data are expressed as the mean ± SEM (n = 3).**(D)** U87 cells were treated with CMPD1 (1 μM) for indicated time. Cell lysates were analysed by Western blotting using indicated antibodies. Representative blots of three independent experiments are shown.

Supplementary Figure S3

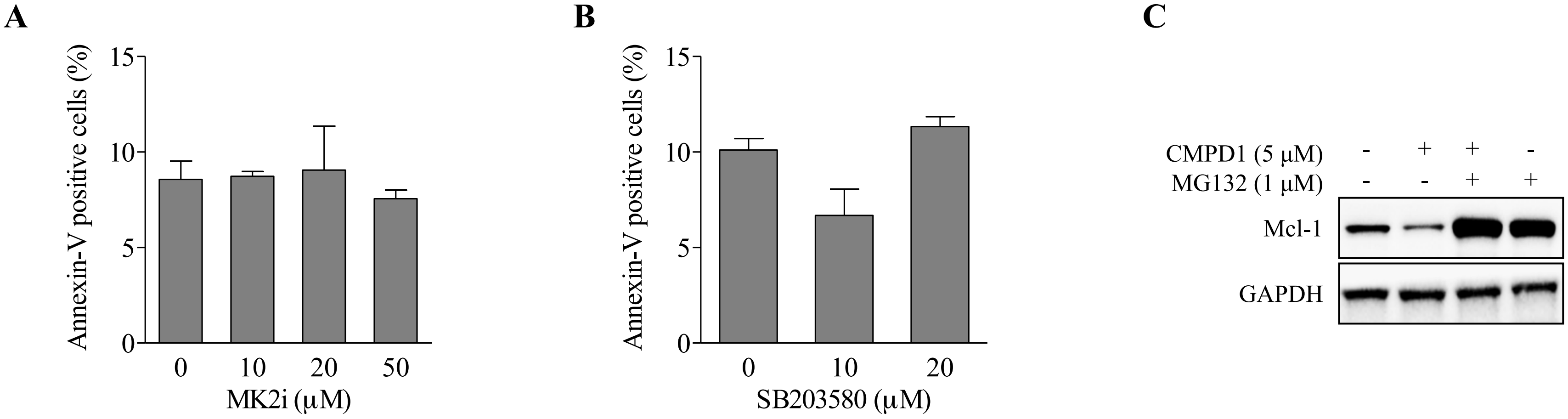

Supplementary Figure S3. (A-B) U87 cells were treated with MK2i **(A)** or SB203580 **(B)** for 48 h and Annexin-V positive cell were analysed with flow cytometry. Data are expressed as mean ± SEM (n = 3). **(C)** U87 cells were pre-treated with proteasome inhibitor MG132 (1 μM; 1 h), then incubated with CMPD1 (5 μM) for 24 h. Cell lysates were analysed by Western blotting using indicated antibodies. Representative blots of three independent experiments are shown.

Supplementary Figure S4

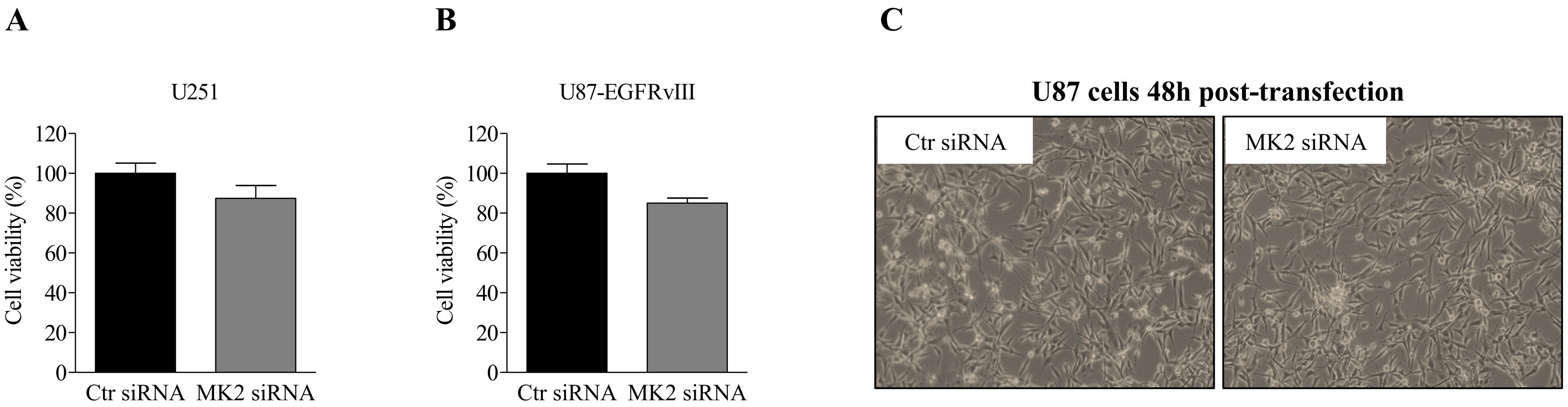

Supplementary Figure S4. (A) U251 cells and **(B)** U87-EGFRvIII cells were transfected with control siRNA or siRNAs targeting MK2 (5 nM) overnight. AlamarBlue cell viability assay was performed 72 h post-transfection. Data are expressed as mean ± SEM (n = 3 - 5). **(C)** Representative image of U87 cells 48 h post-transfection with control siRNA or MK2 siRNA.

Supplementary Figure S5

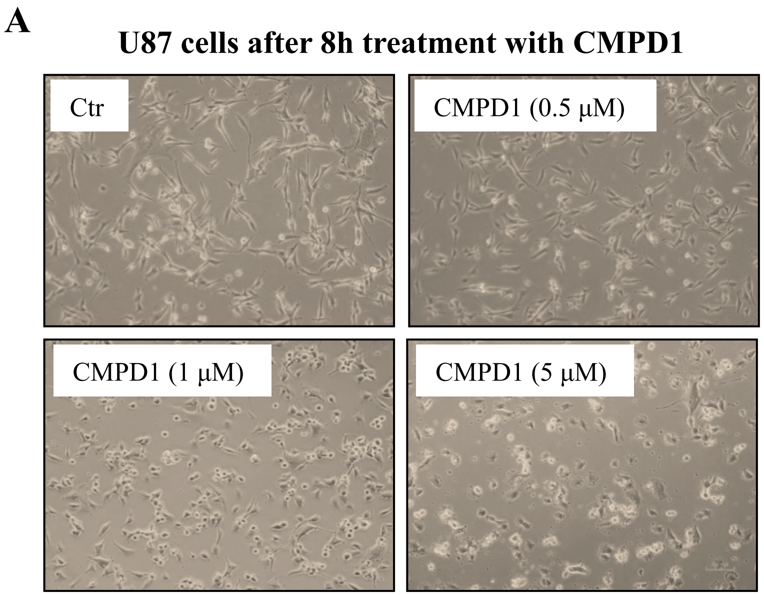

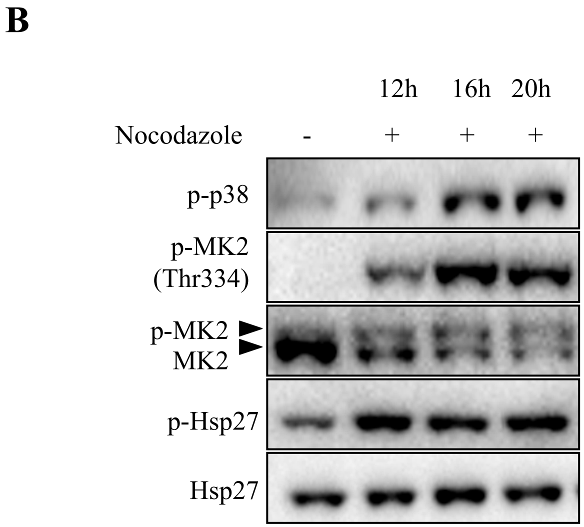

Supplementary Figure S5. (A) Representative images of morphological changes in U87 cells after treatment with CMPD1 (8 h). **(B)** U87 cells were treated with nocodazole (1 µg/mL) for indicated time. Both attached and floating cells were harvested at the 12 h time point, and only floating cells were harvested at the 16 h and 20 h time points. Cell lysates were analysed by Western blotting using indicated antibodies. Representative blots of three independent experiments are shown.

Supplementary Figure S6

A

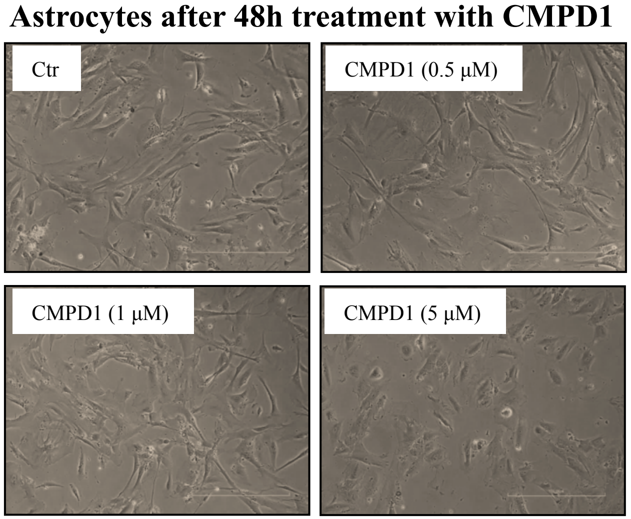

B

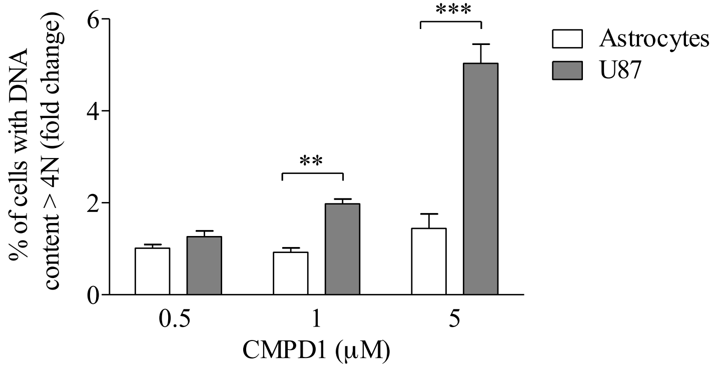

C

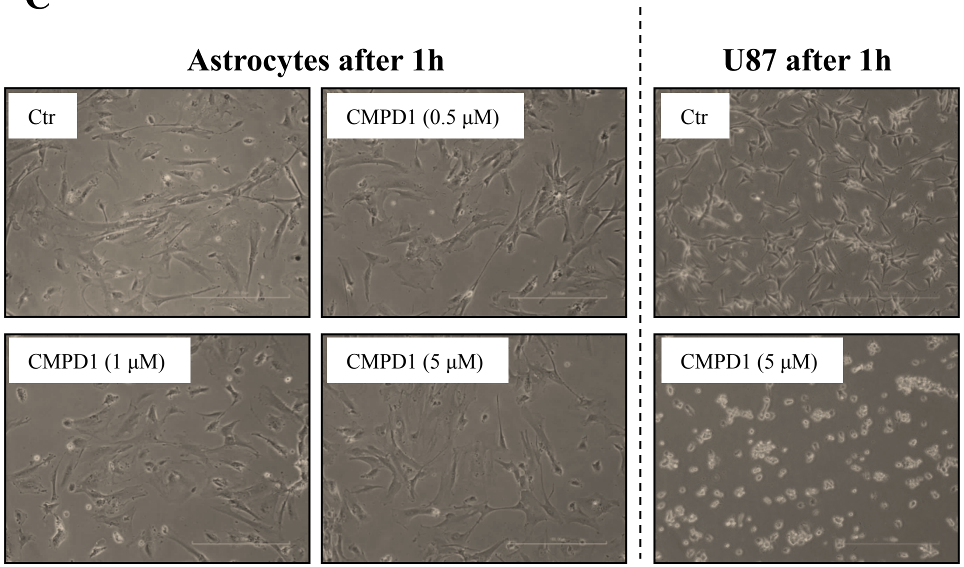

Supplementary Figure S6. (A) Representative images of primary human astrocytes 48 h post-treatment with CMPD1. **(B)** U87 cells or primary human astrocytes were treated with CMPD1 (48 h) and percentage of cells with DNA content greater than 4n was determined using flow cytometry. Data are expressed as mean ± SEM (n = 3; **P < 0.01, ***P < 0.001, 1-way ANOVA followed by Newman-Keuls post-test). **(C)** Representative images of morphological changes in U87 cells *vs* primary human astrocytes 1 h post-treatment with CMPD1.
